# The emergence of human prosociality: aligning with others through feelings, concerns, and norms

**DOI:** 10.3389/fpsyg.2014.00822

**Published:** 2014-07-29

**Authors:** Keith Jensen, Amrisha Vaish, Marco F. H. Schmidt

**Affiliations:** ^1^School of Psychological Sciences, University of ManchesterManchester, UK; ^2^Department of Developmental and Comparative Psychology, Max Planck Institute for Evolutionary AnthropologyLeipzig, Germany

**Keywords:** other-regarding concerns, empathy, normativity, altruism, evolution of prosociality, children, great apes

## Abstract

The fact that humans cooperate with nonkin is something we take for granted, but this is an anomaly in the animal kingdom. Our species’ ability to behave prosocially may be based on human-unique psychological mechanisms. We argue here that these mechanisms include the ability to care about the welfare of others (other-regarding concerns), to “feel into” others (empathy), and to understand, adhere to, and enforce social norms (normativity). We consider how these motivational, emotional, and normative substrates of prosociality develop in childhood and emerged in our evolutionary history. Moreover, we suggest that these three mechanisms all serve the critical function of aligning individuals with others: Empathy and other-regarding concerns align individuals with one another, and norms align individuals with their group. Such alignment allows us to engage in the kind of large-scale cooperation seen uniquely in humans.

## INTRODUCTION

The fact that people are kind to each other is something that most of us take for granted. We see numerous examples of it daily: motorists stopping to let pedestrians cross the street, people holding doors open for others, travelers carrying babies in buggies up staircases, passersby donating a few coins to charities or homeless people, colleagues regularly donating blood. More outstanding examples of prosocial behavior feature regularly in the news, particularly when the helper risks fatal injury to save someone else. Yet, despite their banality, these behaviors are spectacularly unusual when compared to other animals. Outsiders in a society of chimpanzees, for instance, would not expect to receive offers of food or solicitude; rather, they would be fiercely attacked. Even when well-integrated within a group, simple acts such as food sharing come only with begging and harassment (Stevens, 2004; Gilby, 2006). That is not to say that chimpanzees and other species do not engage in mutualistic, and sometimes coordinated, actions with one another. Social life is, for the most part, peaceful. But the fact that humans can interact in a peaceful, coordinated way, with a clear division of labor with unrelated individuals has earned our species the label (granted, a self-made label) of being ultrasocial (Szathmáry and Maynard Smith, 1995; Richerson and Boyd, 1998; Hill et al., 2009).

We see the hallmarks of ultrasociality in our children. They readily incorporate other children into their activities, they share with others (though sometimes under duress), they coordinate their actions with each other, they negotiate meanings, such as rules of games, and they help unfamiliar individuals achieve their goals. How does our ability to cooperate with each other emerge in development and how did it evolve?

In this paper, we address these questions by looking at three key psychological mechanisms. These are the abilities (a) to care about their welfare, (b) to feel with others and to understand how they feel (empathy), and (c) to learn, understand, and enforce norms. We will address the ontogenetic question by reviewing the literature on prosocial behavior in children, and the phylogenetic question by examining findings in our closest living relatives, notably the great apes. The two questions are related: understanding the phylogenetic history of a trait can inform our understanding of its development (Hinde, 1966). The role of empathy in altruism has been discussed before (e.g., Batson, 1991; Hoffman, 2000; de Waal, 2009), but we expand on this by suggesting that empathy is not enough. The ability to empathize can equally lead to antisocial behaviors. Something else is needed to get prosocial behaviors to emerge from empathy. We suggest that this “something else” is an emotional, possibly innate, sensitivity to the needs of others coupled with a motivation toward their welfare. Furthermore, we will argue that norms add enormous complexity and richness to human prosocial behavior, making human prosociality and morality unique in the animal kingdom. We refer to our capacity to respond to the needs of others and to do so normatively as *alignment*, both to other individuals (other-regarding concerns and empathy) and to one’s group (norms). Studying how alignment emerges through development will better enable us to see how the traits constituting it function; looking at our closest living relatives will inform our understanding of its evolution.

To explore the nature of alignment, we will first discuss prosocial behavior and show why preferences for outcomes that benefit others (positive other-regarding preferences) are a necessary feature. The limits of other-regarding concerns in explaining prosociality will also be considered. We will suggest that positive other-regarding concerns motivate behavior that is intended for the improvement of the welfare of others, and discuss the importance of empathy in aligning emotional states. We will then discuss the emergence of norms and their importance in shaping other-regarding concerns. Throughout, we will review the pertinent developmental literature, as well as the comparative literature, to highlight how other-regarding concerns, empathy and a norm psychology could have evolved, as well as how they emerge ontogenetically. Finally, we will briefly speculate on the possible role of alignment that allows humans to be as social as we are.

## PROSOCIAL BEHAVIOR

Prosocial behavior – that is, voluntary behavior that benefits others – seems to emerge very early in ontogeny, with some researchers arguing that it is a biological predisposition (Warneken and Tomasello, 2009a,b). Certainly by 14 months of age, infants help others in simple instrumental ways, such as by handing them out-of-reach objects (Warneken and Tomasello, 2007). During the second year, as children’s cognitive capacities to understand others’ goals and intentions increase, children are able to help others in a wider variety of tasks and in response to a wider array of cues (Rheingold and Hay, 1978; Warneken and Tomasello, 2006; Svetlova et al., 2010). Importantly, early prosocial behavior is not limited to completing others’ action goals. Thus, when 12-month-old infants see an adult searching for an object that they know the location of, they point to direct the adult’s attention to it (Liszkowski et al., 2006, 2008). Given that infants themselves do not gain anything by providing this information, their informative pointing may be considered a prosocial act.

Infants also begin to share objects by the end of the first year and their sharing behavior becomes more sophisticated during the second year of life (Rheingold et al., 1976; Hay, 1979; Brownell et al., 2009; Schmidt and Sommerville, 2011). Children as young as 3 years of age will share rewards and will do so with other children as well as adults (Thompson et al., 1997; Fehr et al., 2008; Moore, 2009; Rochat et al., 2009). Children at about 2 years of age require explicit communication from the recipient to elicit sharing, and even this is not sufficient to prompt much sharing in 18-month-olds (Brownell et al., 2009). Moreover, there are individual differences in how willing 15-month-old infants are to share at a cost to themselves (Schmidt and Sommerville, 2011). Nonetheless, the important finding is that young children share at all, since this is not the rational, self-interested thing to do^1^. Sharing is a particularly interesting form of prosociality because it is costly and because it is important for the evolution of human societies (e.g., Gurven, 2004). It is thus valuable to see how this behavior emerges throughout childhood.

Given the importance of prosocial behavior in humans, the question arises, how did it evolve? The extant species most closely related to humans (i.e., chimpanzees and other non-human primates) do engage in prosocial behaviors. In the wild, they come to the aid of their allies in fights (Harcourt and de Waal, 1992), they console the combatants afterward (de Waal and van Roosmalen, 1979) and they have been observed adopting orphans (Boesch et al., 2010). In experimental settings, they help humans and conspecifics by retrieving out-of-reach objects on request (Warneken and Tomasello, 2006; Yamamoto et al., 2012), opening doors for conspecifics trying to access food (Warneken et al., 2007) and releasing food and non-food items when the recipient acts on the chain holding the reward or signals to the helper (Melis et al., 2010).

However, there is not very strong evidence from the field for food sharing in our closest living relatives. In most cases, in chimpanzees at least, food is shared only under harassment (Stevens, 2004; Gilby, 2006); even mothers will not voluntarily offer novel foods to their own infants unless the infants beg for them (Ueno and Matsuzawa, 2004). In the lab, chimpanzees do not show a preference for outcomes that benefit their groupmates (Silk et al., 2005; Jensen et al., 2006; Vonk et al., 2008). In these studies, chimpanzees were no more likely to choose an option that benefited another chimpanzee and themselves as well (mutualism) than an option that only benefited themselves (selfishness). Remarkably, there was never even a tangible cost, such as giving up some food. Chimpanzees thus seem to be more focused on their own outcomes, and in this sense, behave more like the theoretical *Homo economicus* (Frank, 1987) than do humans. Even in tasks where two subjects working together can both get food, mutualistic cooperation breaks down if the food is not easily divisible (Melis et al., 2006) in contrast to human children who will actively divide rewards after a collaborative task (Warneken et al., 2011). Whether this indifference to outcomes for others holds across different experimental paradigms (Horner et al., 2011) or different species (e.g., common marmosets; Burkart et al., 2007) is a matter of current debate.

Evidence for prosociality in other species raises questions of its own, namely: Are these behaviors similar to and underlain by the same psychological mechanisms as in humans (homologous, that is shared by descent), or do they only superficially resemble human prosocial behavior but are driven by different mechanisms (analogous – similar by selection pressures)? For example, consolation may be more effective at reducing the stress of the consoler rather than the consoled individual and can serve to reduce the likelihood of future attacks (Koski and Sterck, 2007; Koski et al., 2007). Chimpanzees in captivity might hand objects back to experimenters due to prior training and they might remove bolts to open doors and cause food to drop because doing so is an interesting distractor or because the begging and signaling from a conspecific is annoying. Even insects and fish will engage in prosocial behavior – both mutualistic and altruistic (Bshary et al., 2006; Ratnieks and Wenseleers, 2008), and very simple computer programs can appear prosocial (e.g., “generous” tit-for-tit; Wedekind and Milinski, 1996). It is therefore important to not assume that similar looking behaviors are indeed one and the same. Different underlying causes can lead to similar outcomes (for reviews, see Jensen, 2012; Jensen and Silk, 2014).

## OTHER-REGARDING CONCERNS

It is difficult to infer what psychological processes lay behind a behavior such as helping, particularly with naturalistic observations. A single action can have multiple causes. A person seen carrying a suitcase off a train might be doing so to help the traveler reach his or her destination, but the goal might be to run off with the suitcase once on the platform, to impress the traveler with the goal of arranging a date, or even out of spite after the traveler had just struggled to get the suitcase onto the train. An implicit assumption of other-regarding preferences and other prosocial acts is that they are motivated for their effect on other individuals (e.g., Warneken and Tomasello, 2008). However, this need not be the case. People can have ulterior motives. Acting out of self-interest can lead to unintended benefits for others (Adam Smith referred to this as the “invisible hand” guiding markets; Smith, 1776/2005). Selfish motives are particularly relevant in mutualistic interactions – both individuals benefit by working together, but consequences for the partner can be incidental to the actor’s achieving his or her personal goals. Altruistic acts (functionally altruistic in terms of immediate costs and benefits to actor and recipient, not in the biological use of the term which is measured in fitness; West et al., 2007; Clavien and Chapuisat, 2013) are typically clearer demonstrations of actions performed for their effect on another individual, but even these need not be performed for the benefit of the recipient. The other individual can be used as a means to an end. If the end is one’s own happiness (“warm glow” altruism; Andreoni, 1990), then any benefits to others can be unintended side-effects. This is hardly a starting point for truly prosocial behavior.

### POSITIVE OTHER-REGARDING CONCERNS

As stated above, prosocial behavior can arise from a variety of mechanisms and be driven by a range of motivations, but for an act to be truly prosocial (other-regarding) in the sense that the intended goal is the benefit to the recipient, then the actor’s motivation will have to be for the recipient’s welfare (e.g., Batson, 1991). The stance toward the environment that impacts on an individual’s well-being is a *concern* (Prinz, 2007). When the stance is toward the welfare of others, this is an *other-regarding concern*, and it is the motivational basis for truly prosocial behavior. There can be a cognitive component to prosocial behavior, such as recognizing the goals and desires of others, but there must also be a concern that has an emotional consequence – a felt response – for the actor (Nichols, 2004b; Prinz, 2007). Otherwise, there will be no impetus to act on the other’s behalf. Recognizing that someone is in need is not sufficient to lead to helping. For example, seeing a homeless person on the street and recognizing that he needs money does not guarantee that one will give him any loose change, let alone invite him to move into one’s home; some additional motivational force is needed to make one act in a truly prosocial manner. The felt response can be sensitive to the emotions of others, and these are called fortunes-of-others emotions (Ortony et al., 1988). If the emotions are the same as another person’s – e.g., sad for sadness (empathic distress) and happy for happiness (symhedonia) – then they are aligned. Aligned (or congruent) fortunes-of-others emotions will motivate action if seeing someone in distress is distressing and will lead to satisfaction at seeing the welfare of the other improve. The child will seek to remove the source of distress – ideally by helping the distressed other – and will be reinforced by the satisfaction of others. The role of these emotional processes, notably affective resonance and empathy, in social and prosocial behavior will be discussed further in the next section.

While children do help others and share with them, they might not do so out of a concern for their welfare. For instance, they may do so simply because this is what they have been taught to do, along with shaking hands with the right hand and eating with a fork (a point that will be raised in the section on norms). Recent work has begun to address these alternative possibilities, at least with regard to instrumental helping. One line of work shows that young children’s helping behavior is not influenced by parental presence or encouragement, and indeed, is undermined by external material reward, hinting that helping behavior may be intrinsically rather than extrinsically motivated (Warneken and Tomasello, 2008, 2013). Of particular importance to the role of emotions in prosociality, a recent study used physiological measures (pupil dilation) and found that 2-year-old children are not motivated primarily by a desire to help a person themselves (and thus to benefit themselves via reciprocity, an improved reputation or just an interest in engaging with the task), but rather by a desire just to see the person be helped, indicating that toddlers’ prosocial behavior stems from a genuine concern for the other’s welfare (Hepach et al., 2012a).

### NEGATIVE OTHER-REGARDING CONCERNS

Other-regarding concerns are not always aligned. One can feel unhappy at the happiness or fortunate circumstances of others (jealousy and envy), and happy at their unhappiness or misfortunes (schadenfreude; Ortony et al., 1988). Misaligned fortunes-of-others emotions would hardly seem to be ingredients for prosociality. They can form the basis of negative other-regarding concerns, in which the actor is motivated to diminish the welfare of others. Negative other-regarding concerns can also lead to harming behavior, as in the case of sadists being able to grasp how their victims feel but deriving pleasure from their suffering.

Negative other-regarding concerns should not be dismissed simply as the evil twin of positive other-regarding concerns, however. They might also have a place in prosociality. They can motivate punishment, or negative reciprocity, in which harm is returned by harm; this is a powerful disincentive to free-riders who would otherwise exploit prosocial individuals (Boyd and Richerson, 1992; Clutton-Brock and Parker, 1995; Jensen, 2010). In economic experiments such as the public goods game, cooperation – in terms of contributions to a public good – declines quickly with repeated rounds, but with the addition of a punishment option – which is costly for the punisher but provides a benefit for everyone – cooperation can be sustained at a high level (Fehr and Gächter, 2000, 2002; Gürerk et al., 2006). Punishers often feel angry at being cheated (e.g., Pillutla and Murnighan, 1996; Ben-Shakhar et al., 2007) and people (men, at least) will show neurological signatures of pleasure when seeing someone who cheated them apparently receive a painful stimulus (Singer et al., 2006). Negative other-regard, then, can have hedonic value for the punisher, and in the right circumstances, such as when encountering cheaters and free-riders, motivate punishment that is costly at the time it is performed, but can ultimately lead to more prosociality. More generally, punishment is an important means to enforce norms and cooperation, a topic that will be addressed in the section on norms.

It may be the case that other-regarding concerns – both positive and negative – motivate social behavior. Negative other-regarding concerns can be thought of as the spice that accompanies the sugar in everything nice. Our attention will now turn to the emotional substrate for positive other-regarding concerns, both because this has been much more studied in children and because it is of clear and direct value to prosociality.

## EMPATHY

One of the most fundamental sources of prosocial motivation is thought to be empathy, which is an affective response that stems from the apprehension or comprehension of another’s emotional state and is similar to what the other is feeling, and the related process of empathic concern (or sympathy), which is the feeling of concern or compassion for the other (Hoffman, 1981; Eisenberg, 1986; Batson, 1991; Eisenberg et al., 1991). From early in ontogeny, empathy, and especially empathic concern, have been extensively shown to lead to prosocial behaviors and away from antisocial behaviors (Eisenberg and Miller, 1987; Miller and Eisenberg, 1988; Hoffman, 2000). To understand the nature and origins of truly prosocial behavior, therefore, it is imperative to understand the nature and origins of empathy-related processes. This is the aim of the next section, in which we will describe the fundamental components of empathy and empathic concern, and assess the developmental and comparative evidence for those components.

### AFFECTIVE RESONANCE

From the very beginning of life, humans are deeply tuned to the affective states of others. Newborn infants mimic others’ facial expressions and gestures (Meltzoff and Moore, 1977; Haviland and Lelwica, 1987). They also respond by crying when they hear another infant in distress but not when they hear other equally loud sounds or recordings of their own cries (Simner, 1971; Sagi and Hoffman, 1976; Dondi et al., 1999). Such automatic and involuntary emotional contagion persists through the first year (Geangu et al., 2010). From the very start of life, then, humans are deeply connected to others’ internal states, suggesting that this capacity is hard-wired and may be evolutionarily preserved (de Waal, 2008; Decety, 2011). Indeed, mimicry of facial expressions as well as emotional contagion are seen not only in human infants but also in great apes and several other social species (Parr, 2001; Bard, 2007).

Such affective resonance – which is thought to be based on automatic perception-action processes and the mirror neuron system – aligns individuals’ internal states with those of others and, as such, is foundational to countless aspects of social life, ranging from mother-infant bonding, regulating social interactions, and coordinating activities (McDougall, 1908/2003; Blakemore and Decety, 2001; Preston and de Waal, 2002; de Waal, 2008). To take just one example, if one monkey in a group sees something dangerous, such as a snake, he is aroused and produces an alarm call, which produces arousal in his group members and leads the group to move away from the source of danger en masse (Cheney and Seyfarth, 1985). Crucially, though, this phenomenon does not constitute empathy or empathic concern and is not sufficient to motivate prosocial behavior, as discussed next.

### DISTINGUISHING SELF FROM OTHER

When an observer shares the affective state of a target, she experiences a greater or lesser degree of the same or a similar affective state as the other. When this happens and no other mechanisms are at work, the observer will not experience the affect as vicariously induced but rather as being her own and rooted in her own situation, and will thus be motivated to regulate and respond to her own affective state (e.g., by escaping the situation that is causing her distress; Batson, 1991; Eisenberg et al., 2006). In such scenarios, it is unclear what the observer’s motivation would be to respond to the target’s affective state (unless doing so is the only way she could reduce her own arousal). For the observer to identify her affective state as being rooted in the target’s rather than her own situation, the observer must distinguish herself from the other and her own internal states from those of the other. This sense of self as distinct from others is considered essential for affective resonance to become empathic responding (Hoffman, 1976, 2000).

Such self-other distinction is typically tested by mirror self-recognition (MSR), wherein a small mark is surreptitiously placed on an individual’s face and the individual’s behavior in front of a mirror is assessed (Gallup, 1970; Amsterdam, 1972). If the individual shows self-directed behavior (e.g., touching the face), she is thought to have a concept of self as distinct from and in relation to others (see Moore, 2007, for a discussion). Note, however, that other authors have questioned this rich interpretation, arguing, for instance, that MSR indicates an awareness of one’s physical appearance but not a more conceptual awareness of the self (e.g., Suddendorf and Butler, 2013).

In humans, MSR starts by around 18 months of age (Amsterdam, 1972) and has been shown to coincide with the beginning of other-directed and appropriate prosocial behavior (Bischof-Köhler, 1991; Zahn-Waxler et al., 1992a). However, some authors have recently questioned whether the ability to experience empathy requires the kind of explicit, reflective self-knowledge tested by MSR (Davidov et al., 2013). These authors propose that a simpler, more implicit form of self-recognition may be sufficient for empathy, and that this form of self-recognition (based on the infant’s subjective experience of her own sensory perception and self-generated actions) is present from birth (see also Lewis and Brooks-Gunn, 1979; Rochat, 2003). The suggestion is that from the very beginning of life, humans are not only affectively tuned to others’ states but, with the aid of simple and implicit social-cognitive tools, are able to empathize with them. This fundamental capacity stays with us through development and indeed throughout our lives. What changes and improves with development is the ability to mentalize, as well as the child’s knowledge about the world, both of which facilitate the child’s ability to help those in need and respond in more complex situations such as when a victim is absent or when a victim’s immediate cues are inconsistent with the general state of the victim (Davidov et al., 2013).

Support for this proposal comes from recent findings showing that infants as young as 8–10 months do show concern for victims displaying distress, as measured in the infants’ facial expressions, vocalizations, and gestures (Roth-Hanania et al., 2011). Moreover, while the level of empathic concern does not increase between 8 and 16 months of age, infants’ “cognitive empathy” (or attempts to explore and comprehend the others’ distress, akin to a basic theory of mind) does seem to increase gradually over this time period (Roth-Hanania et al., 2011). Other work has also shown that when self-understanding is defined more broadly than MSR (e.g., self-description as seen in the use of own name, use of words such as “me” and “mine”), it relates positively with the degree of empathic concern that children as young as 12 months show for a distressed peer (Nichols et al., 2009).

There is thus some reason to think that empathy could exist even in the earliest stages of infancy. If correct, this proposal may have wide-reaching implications. It implies, for instance, that empathy is a fundamental part of the human make-up. As such, it also suggests that not only affective resonance but also empathy may have deeper evolutionary roots than previously believed. For instance, thus far, the only primate species to reliably show MSR have been the great apes; lesser apes (gibbons) and monkeys seem to differentiate between their own and another monkey’s image in the mirror but fail the formal mark test (de Waal et al., 2005; Suddendorf and Collier-Baker, 2009); this suggests that the capacity for visual self-recognition evolved in a common ancestor of all great apes after the split from the line that led to modern lesser apes (Suddendorf and Collier-Baker, 2009; Suddendorf and Butler, 2013). The conclusion from such findings has been that this prerequisite for empathy is limited to the great apes and humans and is thus relatively recent in our evolutionary history. However, if a more implicit sense of self exists and can fulfill some of the same functions as a more reflective sense of self, then it is possible that many more social species than previously believed have at least one of the prerequisites for empathy (cf. de Waal, 2008), though this certainly need not imply that they necessarily have empathy or empathic concern.

### EMPATHY AND EMPATHIC CONCERN

The abilities to experience affective resonance and to discriminate between self and other are the essential ingredients for empathy, which is the affective response that stems from an apprehension or comprehension of another’s emotional state and is similar to what the other is feeling. However, knowing how another feels via a mechanism such as empathy may be necessary, but is not sufficient, for prosocial behavior motivated out of concern for the welfare of others. This is because *knowing* how the other feels is not the same as *caring* how the other feels. This requires positive other-regard (as discussed above), which has the powerful capacity to bring about empathic concern wherein the observer not only feels as the other feels and identifies the other as the source of the feeling but also *cares* what happens to the other and is therefore *motivated* to enhance the welfare of the other. Notably, this means an alignment not only of the observer’s and target’s affective states but also of their goals and motivations: Both the observer and the target are invested in and affected by the target’s welfare and both are thus motivated to improve it (Hepach et al., 2013; Vaish and Tomasello, 2014). We now have the ingredients in place for empathic concern (or sympathy), which provides a fundamental motivational force for prosocial behavior.

Decades of research show that toddlers and young children respond with empathic concern toward others and that this empathic concern motivates prosocial behavior. Typically, infants see a person (parent or stranger) experience a negative situation (bumping her knee against a table, for instance) and overtly showing pain, distress, or sadness. In such situations, infants as young as 14 months of age show concern in their facial and vocal expressions and often attempt to alleviate the victim’s distress by comforting, helping, or sharing with her (Eisenberg et al., 1989; Zahn-Waxler et al., 1992a,b; Svetlova et al., 2010). Moreover, the empathic concern that infants and toddlers show in these situations correlates positively with their prosocial behavior toward the victim (Hoffman, 1982; Eisenberg and Miller, 1987), indicating that empathic concern serves prosocial motives from early in ontogeny. This work provides evidence for an early capacity to experience empathic concern stemming from affective resonance whereby children automatically share the victim’s affect, distinguish between self and other, and, in conjunction with positive other-regard, experience empathic concern for her.

Whether empathic concern is present even during the first year (with the aid of implicit self-recognition capacities) is an open question. As noted earlier, some recent work suggests that infants as young as 8–10 months show concern for others displaying distress (Roth-Hanania et al., 2011; see also Nichols et al., 2009). Certainly by the second year, human children experience other-directed empathic concern and this concern motivates their prosocial behavior toward those in need.

The story is far less clear when it comes to empathic concern in the great apes. As discussed above, a sense of self is present in the great apes, but other-regard may or may not be, making it difficult to formulate a clear hypothesis about whether empathic concern could or could not exist in these species. A recent experimental study aimed to directly test whether empathic concern motivates prosocial behavior in the great apes (Liebal et al., 2014; based on a similar study with children by Vaish et al., 2009). In this study, subjects saw a conspecific being either harmed (a human experimenter stole the conspecific’s food) or not being harmed (no food was stolen from the conspecific). Subsequently, subjects had the opportunity to provide help to the conspecific in a new task. The logic was that if subjects experience empathic concern for a conspecific who is harmed (versus one who is not harmed), then this concern should motivate their subsequent prosocial behavior toward the conspecific. The results did not support this hypothesis, as apes helped their conspecific equally if he had previously been harmed than if he had previously not been harmed, suggesting that their prosocial behavior was not motivated by empathic concern. However, much more work is needed to rule out alternative explanations. For instance, perhaps the harm situation was simply not serious enough to elicit concern in the subjects. Equally, perhaps apes do experience concern for others but this concern does not necessarily translate into helping behavior of the kind measured in the study; instead, perhaps apes would be more likely to groom or console a conspecific for whom they felt empathic concern (Liebal et al., 2014).

Empathy has been attributed to numerous other species as well. For instance, recent studies, in line with decades-old research (Church, 1959; Rice and Gainer, 1962), conclude that mice and rats are empathic because they exhibit heightened pain responses after seeing conspecifics in pain (Langford et al., 2006) and altruistically open doors to release conspecifics trapped in tubes (Bartal et al., 2011). However, we must be cautious in our interpretations here because there is no reason to believe that empathic concern, or even more “primitive” emotional contagion, are involved. Like rats, ants show releasing behaviors, and there is nothing to suggest that an emotional mechanism tuned to the welfare of others is involved there (Nowbahari et al., 2009; Vasconcelos et al., 2012). Furthermore, even if the rats did experience emotional contagion, the “helping” rat did not have an alternative, such as escaping to avoid the distressing stimulus of a stuck rat, a key element of empathy–altruism studies on adults (e.g., Batson et al., 1981). Moreover, the helper might simply be curious, especially given that after the rats have opened the tube door, they often go inside and explore it, or they might have been seeking social contact (Silberberg et al., 2014). These issues must be given special consideration when studying animals most akin or familiar to us due to our tendency to anthropomorphically project human characteristics onto other species (Wynne, 2004; Barrett, 2011).

Overall, then, humans demonstrate empathic concern from as early as the second year of life and such empathic concern motivates their prosocial behavior toward victims. However, the jury is still out as to whether empathic concern may occur even earlier in human ontogeny as well as about whether it occurs at all in other species, including our closest living relatives.

### BEYOND AFFECTIVE RESONANCE

Our discussion of empathic concern has thus far focused on empathic concern grounded in the fundamental capacity for affective resonance. That is, when an observer attends to a target in pain or distress, he experiences resonant affect that, with the aid of self-other discrimination and other-regard, can become empathic concern. But humans (at least) have higher cognitive capacities that allow us to experience empathic concern even without affective resonance. Perhaps most prominently, even when we have no perceptual access to a target’s emotional state, our ability to take others’ perspectives allows us to imagine how the target might be feeling and perhaps experience empathy as a result. As noted earlier, knowing how another feels (either through affective resonance or perspective taking) is not sufficient to elicit concern or prosocial behavior. However, in conjunction with positive other-regard, imagining or understanding how another feels can enable us to experience empathic concern for the other (Feshbach, 1978; Hoffman, 1984; Eisenberg et al., 1991; Batson et al., 1997; Ruby and Decety, 2004).

Interestingly, even when we do experience affective resonance in response to overt perceptual cues, we are able to use our contextual appraisal abilities to modulate our empathic concern as appropriate (e.g., Lamm et al., 2007a,b). For instance, if adult participants are made to believe that the hands they see in painful situations have been anesthetized, their empathic concern is significantly dampened compared to when participants do not believe the hands are anesthetized (Lamm et al., 2007b)^2^. Such processes act as *top-down* generators and modulators of empathic concern, adding tremendous scope and flexibility to our empathic system by ensuring that we are able to respond empathically – and thus prosocially – in diverse situations and toward diverse victims (Hoffman, 2000; Decety and Lamm, 2006; Singer and Lamm, 2009; Decety, 2010; Vaish and Warneken, 2012).

Recent work provides evidence for this extended scope and flexibility even in young children’s empathic concern. One line of work has explored whether children can experience concern even in the absence of any perceptual access to a victim’s distress. In one study, 6-year-old children who observed an adult being harmed (another adult destroyed her artwork) showed expressions of concern for her even though she did not display any distress (Hobson et al., 2009). A further study found that even 18- and 25-month-old children showed greater facial concern for an adult who was harmed but displayed no distress than for an adult who was not harmed. Moreover, when the adult subsequently needed help, children were more prosocial toward her if they had previously seen her being harmed than not being harmed, and individual children’s concern while seeing the adult being harmed correlated positively with their subsequent prosocial behavior (Vaish et al., 2009; see also Vaish et al., 2010b). These studies show that human empathic concern is multi-determined (evoked in response to several types of cues – both emotional and situational) from early in development.

In a second line of work, researchers have begun examining whether contextual appraisal plays a role in children’s empathic concern. In one study, 3-year-old children showed reduced concern and subsequent prosocial behavior toward a “crybaby,” i.e., a person who was considerably distressed after being very mildly inconvenienced, than toward a person who was similarly distressed after being more seriously harmed (Hepach et al., 2012b; see also Leslie et al., 2006; Chiarella and Poulin-Dubois, 2013). Thus, young children’s empathic concern is impacted by not only the presence or absence of distress cues from a person but also the contextual cues surrounding the distress.

To sum up, top-down processes such as perspective-taking and contextual appraisal add scope and flexibility to humans’ empathic concern from an early age (certainly by the middle of the second year). This allows even young children to, on the one hand, align their affective states with those of others in a broad array of situations and in response to various types of cues, and yet, on the other hand, have the flexibility to modulate their empathic concern so that they can direct their concern and prosocial behavior toward those who truly need it. This sophistication in early empathic concern speaks to the complexity of human social interactions and the vital role played by empathic concern in allowing for and regulating such interactions.

Whether empathic concern with such scope and flexibility may be available to any other species is as yet unknown. Before tackling this question, however, it would seem much more fruitful for future work to examine whether or not empathic concern through affective resonance exists in other species. This is because if any form of empathic concern is likely to exist in other species, we think it is most likely to be empathic concern that arises out of affective resonance – given that the foundations for such empathic concern lie in the automatic, perception–action mechanisms that all social species share (Preston and de Waal, 2002). As mentioned above, the first experimental test of empathic concern in the great apes failed to find evidence for such empathic concern (Liebal et al., 2014), but much more work is needed to draw firm conclusions. More multi-determined and flexible forms of empathic responding require higher cognitive (and emotional regulation) skills that the great apes may or may not possess (see Call and Tomasello, 2008; Koski and Sterck, 2009). Our prediction here is that if any empathic concern exists in species other than humans, it will be the most fundamental kind – evoked by overt emotional signals and via affective resonance – and that much more complex and sophisticated forms of empathic concern may well be unique to humans.

So far, we have been concerned with alignment on the interpersonal level, that is, alignment with others through empathy and empathic concern. Humans also interact on the impersonal level (e.g., in third-party interactions), that is, they align with their group through social norms; this is the topic of the next section.

## NORMS

Human infants are born in a world replete with normativity. Thus from early on, the young learner needs to make sense of human social interactions in a given cultural context and discern which actions (e.g., hitting someone else) are generally prohibited or prescribed (and thus come with binding force or “oughtness”) and which actions (e.g., petting a dog) are merely idiosyncratic and thus not subject to norms. But when and how can the young learner make and understand this distinction? And how do empathy and other-regard interrelate with children’s developing norm psychology? In what follows we will first describe some important theoretical and conceptual aspects of social norms and then look at evidence suggesting that even young children have a robust understanding of social norms.

There are many different ways to describe social norms, but perhaps the most crucial features are their *binding force* – that is, people “should” or “ought to” perform certain actions in certain contexts, thus have reason to act in certain ways (Searle, 2001) – and their *generality* – that is, norms apply to all participants of a social practice alike (Nagel, 1970). Thus, we have *normative expectations* about how people ought to act in certain situations in our cultural group (Chudek and Henrich, 2011). An important consequence is that social norms help and even urge us to align with our group members, and so they are essential to social order – at least by making others’ behavior more predictable, albeit not necessarily more cooperative or moral (Elster, 1989). For example, codes of honor or dress norms (e.g., wearing ties at office) need not make people more cooperative, but one can predict what is likely to happen in a certain situation. Moreover, there are many conflicting norms, for example, about how to allocate resources in a society (e.g., in egalitarian vs. utilitarian ways), and these conflicting norms are frequently obstacles to compromise at different levels within a society. Here, we suggest, is the important role of human other-regard and empathetic competencies in both the ontogeny and phylogeny of human norm psychology and morality. But before examining the interrelations between other-regard, empathy, and normativity, we first need to know what young children actually understand about social norms as entities that come with binding force and generality – not least because morality is essentially based on norms (Piaget, 1932).

### NORMATIVE FORCE AND GENERALITY

There is a rich literature on children’s moral knowledge – starting with Piaget’s (1932) pioneering work – that is, their judgment of norm transgressions in hypothetical scenarios, suggesting that by 3–4 years of age, children make robust distinctions between existing conventional norms (e.g., proper classroom behavior) and existing moral norms (e.g., the prohibition to hit someone else). In particular children have been repeatedly shown to categorize moral transgressions as more severe, less dependent on context, less contingent on authority, and more deserving of punishment than conventional violations (Smetana, 1981; Turiel, 1983; Killen and Smetana, 2006; Killen and Rutland, 2011).

The focus here, however, is on children’s normative judgment in action, that is, on their understanding of the force and the generality of norms in social interactions. The reason for this is twofold. First, normativity is fundamentally about human actions and therefore about practical norms that give people (normative) reasons to act in certain ways (distinct from reasons to think in certain ways; Wallace, 2011); thus, the question of whether children understand the force and generality of norms can be answered best by assessing whether they, as unaffected observers, demand from third parties to act in certain (prescribed) ways^3^. Second, looking through an evolutionary lens, it is primarily “adaptive” social actions that are relevant for natural selection (Vaish and Tomasello, 2014) such that some kind of coordinative, cooperative, and moral behaviors made some hominin ancestors, or groups, more successful than others (Alexander, 1987; Krebs, 2008; Tomasello et al., 2012). In what follows, we will look at children’s enforcement of conventional and moral norms and the importance of these types of norms for processes of alignment.

### CONVENTIONAL NORMS

We live in a world of traditions, customs, and existing social practices, so it can be easy to forget that norms are essentially socially constructed facts that could have been different (i.e., they are arbitrary). We typically follow conventional norms and this leads to alignment with one’s group. For instance, we drive on a particular side of the street, dress in certain ways in certain contexts, or greet each other in certain ways. However, mere norm adherence does not tell us whether individuals are committed to the norms or just intend to avoid sanctions. Enforcing (often arbitrary) conventional norms as an unaffected observer, however, not only fosters group-wide alignment, but also entails some “impersonal prosociality” on the part of the enforcing group member as it indicates that the individual cares about the group’s values and ways of doing things *per se*, not just about whether they serve the self (Rossano, 2012; Schmidt and Tomasello, 2012). Hence, our understanding of the development of prosocial behavior can be greatly enriched by our understanding of the emergence of conventional norm enforcement.

A recent line of research has used an action-based approach to assess children’s normative understanding. Investigators put children into social situations in which different types of third-party norm transgressions occurred (typically committed by puppets). Thus, it was possible to examine children’s understanding of the force and the generality of norms by dint of their spontaneous (verbal and behavioral) interventions against norm transgressors.

This line of research has found that by 2–3 years of age, children criticize and protest conventional norm violations, for instance, when third parties break the rules of a simple game; in particular, 3-year-olds often use normative language (e.g., “This is how it is done!”) when reprimanding others (Rakoczy et al., 2008). Moreover, children preferentially enforce novel conventional norms they learn from adults rather than from peers, and from reliable versus unreliable models (Rakoczy et al., 2009, 2010). Interestingly, young children do not need explicit teaching, ostensive cues (Gergely and Csibra, 2006; Csibra and Gergely, 2009, 2011), or normative language by the model to infer that an act is normative and culturally relevant: Schmidt et al. (2011) found that 3-year-old children learn novel conventional norms by mere incidental observation of a confident adult that does not perform a game-like action for the child’s benefit. Hence, young children are not only adept at following conventional norms, they even enforce them when third parties transgress, thus providing evidence for an early impersonal prosociality.

### MORAL NORMS

Alignment with group members occurs not only by means of conventional norms but also moral norms (e.g., against harming one another), many of which help sustain human cooperation and suppress individuals’ self-interest (Joyce, 2006; Krebs, 2008). As with conventions, group members not only follow these norms, but they also enforce them against third-party transgressors. On a functional level, enforcement of such norms is considered prosocial or costly because the enforcer provides the group with a benefit but risks retaliation (Fehr and Fischbacher, 2004; Kurzban et al., 2007). Some norms (often considered moral) carry more normative weight than others – that is, some violations cause particularly strong emotional reactions in unaffected observers (Nichols, 2004a; Rossano, 2012). And the normative weight of a given norm is adjusted by other-regard and empathy (observers need to be moved at all by some action), and by the collectivistic and normative understanding (e.g., “One should not harm others”) that feeds back into the process and reinforces other-regard and emotional reactions to norm violations in a cultural context.

Recent work has found that by 3 years of age, children protest violations of moral norms, such as those against destroying or throwing away others’ property (Vaish et al., 2010b; Rossano et al., 2011). Preschool-aged children also direct less helping toward harmful individuals and prefer (verbal) punishment to be directed at immoral individuals rather than at victims (Vaish et al., 2010a; Kenward and Dahl, 2011; Kenward and Östh, 2012). Another recent study shows that 3- and 5-year-old children will punish puppets that violate a moral norm (theft) by making the stolen object inaccessible to all individuals, or restoring them to the original owner when that option exists (Riedl et al., in preparation).

Typically, moral norms are considered wide in scope and thus applicable to virtually all people (Turiel, 1983; Korsgaard, 1996; Scanlon, 1998), whereas conventional norms are narrow in scope and thus applicable only to those who (implicitly or explicitly) agreed on them (Searle, 1995; Diesendruck and Markson, 2011). The nature of this distinction is highly debated, with some arguing for a categorical divide and conceptually distinct domains (Turiel, 1983) and others suggesting a distinction between norms accompanied by strong feelings (e.g., norms prohibiting harm, but also disgusting actions) and norms without or with less emotional involvement (Haidt et al., 1993; Nichols, 2002, 2004b; Kelly et al., 2007). What is clear, however, is that moral and conventional norms are distinct along at least some dimensions.

Indeed, a wealth of interview studies have shown that children and adults show systematically different response signatures when confronted with hypothetical vignettes about paradigmatic moral versus paradigmatic conventional norm violations (Smetana, 1981; Turiel, 1983; Tisak and Jankowski, 1996; Turiel, 2002, 2006). Most importantly, moral transgressions are categorized as more severe, more deserving of punishment, and less contingent on authority or context. But how do young children understand the scope of moral versus conventional norms? Who *ought* to follow these norms – any third party or ingroup members only? A recent study investigated this question and found that 3-year-olds show systematically different patterns of norm enforcement in response to violations of paradigmatic conventional and moral norms: Children protested violations of moral norms (against destroying another’s property without any obvious reason) equally for ingroup and outgroup individuals, but they enforced conventional norms (about simple game rules) for ingroup members only (Schmidt et al., 2012). Thus, children recognized that conventional norms are group-specific in nature and therefore apply only to ingroup members who can be expected to respect them.

The space of morality, however, is not confined to people having obligations to perform or refrain from certain acts. People also have rights that are mutually recognized (Turiel, 1983; Helwig, 1997; Killen and Smetana, 2006). And the key feature of a right or entitlement is that they are inherently linked to obligations by others and hence create normative constraints on others’ conduct (Rainbolt, 2006; Searle, 2010): When some right-holder R is entitled to do something (e.g., to use someone’s property), then others are obligated not to interfere with R’s entitlement. A recent study examined young children’s understanding of rights in different contexts and found that 3-year-olds, as unaffected observers, enforce and defend a right-holder’s legitimate entitlements (e.g., being granted permission to use an object by the owner of that object) against someone who threatened the right-holder’s entitlements, for instance, by taking away an object (Schmidt et al., 2013).

Fairness – for instance the principle of equality – is particularly important in discussions of conventional and moral norms (Rawls, 2001) and has long been a topic of interest in the study of moral development focusing on distributive justice (Piaget, 1932; Hook and Cook, 1979). Expectations about fairness appear early in development and may be linked to prosociality. For instance, Schmidt and Sommerville (2011) found that 15-month-old infants expect resources to be distributed equally, and importantly, that these third-party expectations are closely linked to infants’ own other-regarding sharing behavior: Infants who share altruistically (part with a toy they prefer) are more concerned about fairness than infants who share selfishly (part with a toy they do not prefer). This interrelation between fairness and other-regard was found for costly sharing behaviors in 12- and 15-month-old infants, but not for *prima facie* less costly instrumental and informational helping behaviors (Sommerville et al., 2013).

The ultimatum game is the most widely used tool for probing fairness preferences in adults (Güth et al., 1982). In this game, one “player,” the proposer, has an endowment that can be shared with the second player, the responder. If the responder accepts the offer, both get the proposed division, but if he or she rejects it – out of a sense of perceived unfairness – both get nothing. Four-year-olds make fair offers in response to the threat of rejection (Takagishi et al., 2010) and this strategic decision-making continues to improve between 6 and 14 years (Steinbeis et al., 2012). Of particular importance is the rejection of unfair offers due to disadvantageous inequity aversion (e.g., Fehr and Schmidt, 1999; Falk and Fischbacher, 2006). Five-year-old children do reject unfair offers in a reduced form “mini” ultimatum game in which there are paired choices (e.g., 50/50 vs. 80/20). Unlike adults (Falk et al., 2003), however, the children do not show sensitivity to outcomes, nor even to the intentions of the proposer, but rather attend to a particular sharing norm, namely that parity constitutes fairness (Wittig et al., 2013). The details of what constitutes a fair offer is not universal – offers of 50% are not found in all cultures, and people in different cultures do not always punish offers that depart from this (e.g., Henrich et al., 2006). However, in ultimatum game studies, children will punish personally unfair offers (disadvantageous inequity aversion), even though it is costly to do so (for other examples, see also Murnighan and Saxon, 1998; Bereby-Meyer and Fiks, 2012). Surprisingly little is known about how children in different societies understand fairness (e.g., Rochat et al., 2009; Zebian and Rochat, 2012; House et al., 2013)^4^.

In sum, the research reviewed here suggests that young children are highly motivated to seek out social norms, to acquire them, and perhaps most importantly, to enforce them as unaffected observers. They apply both the normative force and the generality of norms. Still, they make important distinctions and apply norms selectively depending on context and scope. And so they also appreciate the conventionality of many norms (e.g., the group-specificity of conventional norms). Young children’s normative learning is guided by rational principles as they take into account social-pragmatic and contextual cues (e.g., they preferentially learn from competent models). Hence, these findings from diverse domains of normativity suggest that young children already have a basic understanding of important properties of our normative reality.

### LEARNING NORMS

In children, normative learning most likely capitalizes on early infant–caregiver interactions, including ritualized behaviors, sharing affective states in joint activities, and reciprocal imitation (Tomasello et al., 2005; Rochat, 2007; Rossano, 2012). Importantly, infants and young children can also use second- and third-party emotional appraisal (i.e., external sanctions and reward) as a compass for what others (their culture) understand as normative. For instance, a caregiver might show a strong emotional response when one child hits another (Smetana, 2006). However, the young learner actively makes sense of these situations with capacities for other-regard, empathy, and normativity, so this is not a unidirectional process of cultural–emotional conditioning (Prinz, 2007), but an interaction of the child’s predispositions (i.e., empathic concern and norm psychology) and the respective normative-cultural context (Turiel, 1998; Smetana, 2006).

Once children have aligned with their group and internalized the group’s norms, they may also apply personal emotional appraisal (i.e., internal sanctions or reward) such that they may even judge their own transgressions negatively and punish themselves through guilt and shame, and may reward themselves for having lived up to a social norm via pride (e.g., Zahn-Waxler and Kochanska, 1990; Barrett et al., 1993; Tangney et al., 2007). Such emotions are important for self-regulation, serve as motivations to act normatively in the future (Kochanska and Aksan, 2006), and help children follow the norms of the group more generally. (It remains to be seen whether other animals, great apes in particular, experience self-conscious emotions; dogs will show an anticipation of punishment that can be confused with – but is not – guilt; Horowitz, 2009.) Beyond experiencing self-conscious emotions such as guilt, young children also show a preference for and distribute more resources to transgressors who display guilt than those who display no guilt, suggesting they understand the important appeasement functions that guilt serves after norm violations (Keltner and Anderson, 2000; Vaish et al., 2011). It is important to note here that although third-party and personal emotional appraisals are a vital aspect of normativity, they can only explain children’s adherence to norms, not their motivation for enforcing norms, since these require the alignment mechanisms based on other-regard and empathy discussed above.

### THE EVOLUTION OF NORMS

The important evolutionary question is when in human history did the key mechanisms for normativity – namely their binding force and generality – evolve. At present, there is no evidence that primates have anything resembling norms. They do follow sanction-based “rules” in their groups, such as “subordinate individuals do not take food away from dominants,” but there is nothing binding or general about these. Individual learning and fear of retaliation is sufficient. Primates have been said to have a “respect for possession” in which dominant individuals will not take food from subordinates (Kummer and Cords, 1991), but this is, of course, not a normative notion, and a rather crude analogy to the normative institution of ownership in humans. In chimpanzees, for instance, subordinates will vocalize loudly in response to food theft, calling the attention of the group, which might chase or threaten the food “thief.”

There has been some suggestion that some individuals (dominants) in non-human primates will “police” their social groups by intervening in fights (Flack et al., 2006; von Rohr et al., 2012). However, in the only experimental test of third-party punishment, dominant chimpanzees did not punish a third-party “violation” – namely one individual taking food – even when the “victim” was genetically related to the impartial observer and the observer was dominant to the “thief” (Riedl et al., 2012)—even though they did “punish” by collapsing a food table when their own food was taken (Jensen et al., 2007b). Any policing or punishment in non-human primates and other animals does not need to appeal to normativity or impersonal group concerns. Yet, despite this, groups of animals, including chimpanzees, can exhibit regional differences in behavior – traditions – that some authors refer to as cultures (e.g., Whiten et al., 1999). They do learn socially from their groupmates in a way that might support the emergence of culture, or something akin to culture (e.g., Horner et al., 2006; Hopper et al., 2011; van de Waal et al., 2013), but there is considerable debate as to whether social learning is even necessary (e.g., Tennie et al., 2009; Langergraber et al., 2010). Chimpanzees and other non-human primates, then, might not have the evolutionarily more recent elements for normativity, but they have some capacity for social learning that is certainly essential. When and how the key elements of normativity evolved remains an open question (for one possibility, see Tomasello et al., 2012).

## DISCUSSION

We have argued that two forms of social alignment – alignment with other individuals (interpersonally) and with the group (impersonally) – form the bases for human morality and prosociality. We align ourselves with other individuals by way of empathy and other-regarding concerns, especially empathic concern, which allow us to feel with and for others. And we align ourselves with the group by way of normativity. These two forms of alignment are intricately linked, and they together give rise to uniquely human forms of prosociality and cooperation, both at a small scale, namely families and tribes, and at a large scale in groups of unrelated strangers. Empathy, other-regarding concerns, and norms lead to alignment, and group-wide alignment on interpersonal and impersonal levels is not merely an outcome, but also feeds onto individual human psychology. This, in turn, changes the social dynamics of human group life (in stark contrast to, say, chimpanzee group life). Though some other species – in particular the great apes – might align themselves interpersonally with other individuals via affective resonance, they might not be able to do so via empathic and other other-regarding concerns, and there is no evidence to suggest that they align themselves impersonally with the group via normativity. Human ultrasociality evolved, but it is not yet clear when in our history we displayed the first signs of our “better nature.”

To further our understanding of the development and evolution of ultrasociality, future work will need to examine these alignment processes in more depth. The work on the origins of other-regard, for instance, is rather limited. Among children, studies are only now emerging that show that young children genuinely care about the welfare of others (Hepach et al., 2012a, 2013), and this work has only explored simple instrumental helping situations. Whether such other-regard is present across diverse prosocial contexts and when it emerges in development are vital questions to answer if we are to understand the nature of this fundamental alignment process. Equally, it is important to explore this process in the other great apes using a similar method as with children, which will help establish whether the uniqueness of human ultrasociality stems from this most basic alignment mechanism. Much more work is also needed to establish the role (or lack thereof) of empathy and empathic concern in the prosocial behavior of the great apes. Although we know a great deal about empathic processes in infants and young children, systematic investigations with the great apes are severely lacking, and the little work that exists, though suggestive, is open to alternative interpretations (e.g., Liebal et al., 2014). As more evidence emerges, the picture of the interpersonal alignment processes in humans and other species will become clearer and will help shape further hypotheses about the shared versus unique aspects of human prosociality.

How empathic, other-regarding, and normative capacities evolved – whether together or independently – is also an open question. We suggest that they are mutually dependent both in ontogeny and in phylogeny. Young children care a lot about others in interpersonal and impersonal (i.e., third-party normative) interactions, and they care about norms for the norms’ sake – most clearly evidenced by their enforcement of totally arbitrary conventional norms, such as game rules (Schmidt and Tomasello, 2012). Most generally, other-regarding concerns and empathy help humans cooperate in such a way as to create, learn, understand, and maintain norms. In turn, norms help to structure and determine contexts in which other-regarding behavior and empathic concern occur.

The capacities for empathy and other-regard make it more likely for some norms to emerge and to persist. These are, for example, norms that have to do with cooperation, such as norms of reciprocity, norms against harm, norms regarding justice (e.g., in resource distribution) and the like. For these norms, suppression of self-interest and some concern for other conspecifics’ welfare is crucial. Thus, children’s early other-regard and empathy are morally relevant in the sense that they help them learn and understand cooperative norms, and to be motivated to follow and enforce these norms. The direction of this process is from interpersonal (other-regard, empathy) to impersonal (normativity). One consequence of this process would be that human infants acquire norms of distributive justice (in particular fairness as equality) early because of their concern for others’ well-being and their early first-party and third-party experience with fairness situations (e.g., desiring resources oneself and observing others desiring resources; see Geraci and Surian, 2011; Schmidt and Sommerville, 2011).

Other-regard and empathy also have an impersonal dimension. They help the young child to identify with the group and to be emotionally committed to the group’s values and norms (Tomasello, 2009; Rossano, 2012; Schmidt and Tomasello, 2012). This then strengthens motivations to care about the group’s norms and thus not only to follow them, but also to defend and enforce them in interpersonal and impersonal interactions. Importantly, this impersonal dimension not only leads to punitive behaviors for norm violations, but also constructively fosters conformity, for instance, by teaching others the group norms. One key point here is that the norms apply to the group. What constitutes a group can be arbitrary. For instance, in the classical “minimal group paradigm,” group assignation such as preference for certain artists, can lead to in-group favoritism (Tajfel et al., 1971). In addition to increased cooperation within an arbitrarily created group, it can also lead to increased punishment of norm violations within the group, but not across groups (Shinada et al., 2004; Bernhard et al., 2006; Goette et al., 2006). Parochialism has also been demonstrated in children on the basis of which school class they belong to (Fehr et al., 2008). It would seem that the general direction of this process is from the impersonal to the interpersonal, and children’s propensity to enforce different types of norms in different contexts is paradigmatic of this process.

Norms go far in shaping which behaviors are appropriate in which contexts, and moral norms (in particular those related to harm) have special normative weight. Even so, there can be norms for everything, and conduct rules for helping others and preventing harm are not universal. The foundations for uniquely human ultrasociality thus comes from the combination of an emotional, possibly innate, sensitivity to the needs of others, coupled with a motivation toward their welfare. Norms systematize, standardize, and contextualize for the group which prosocial (or antisocial) behaviors are expected, when, and toward whom.

To explore the role alignment plays in interpersonal and normative behavior, future studies can pit these alignment processes – alignment with an individual versus a group – against each other to see how children resolve them. For instance, there may be situations in which empathic concern is likely to motivate one course of action whereas social norms might prescribe another. As another example, norms for how one ought to behave toward ingroup members as opposed to members of other groups can come into conflict (see also Killen and Rutland, 2011), especially when what constitutes a group is fluid (an ingroup member can be anyone from a child’s class, but can also be anyone of the same gender regardless of which class he or she is in). Importantly, these research questions would need to be applied across various cultures to explore the importance of norms for interpersonal alignment. While it would not be possible to test norms in non-human animals in the same way as in children, it would be worth investigating whether other species are sensitive to individuals who align with others or the group and those who do not, such as by pursuing self-interests ahead of those of others. This work could be done on our closest living relatives, as well as by comparing species that have complex social interactions versus those that do not (e.g., wolves vs. foxes) or cooperative breeders and non-cooperative breeders (e.g., meerkats vs. banded mongooses). Future work could also explore how sensitive individuals are to cues of alignment from others. For example, if another child shows a concerned look for a child (or a third party), or signs of shared joy, a child who is sensitive to interpersonal alignment should be more likely to engage in mutualistic or prosocial acts toward that person than toward someone who shows no emotional cues of alignment, or shows signs of misalignment. Whether other species even have the appropriate signals is an open question. Certainly dog owners will recognize concerned looks in their dogs; it is not clear whether dogs would also recognize and use these looks to cue alignment.

It is possible that mutualistic, coordinated interactions – among interdependent individuals – explain the first step toward ultrasociality, followed then by inter-group competition, which led to the formation of norms (Tomasello et al., 2012). We suspect that the core elements for ultrasociality arose first in small-scale, interdependent interactions, such as dyads (face-to-face and two individuals in a collaborative activity) and in small groups (e.g., observing two people interacting). These small-scale interactions were greatly facilitated by, and thus gave rise to, the ability for individuals to align their emotions (empathy), as well as their goals (joint intentionality; e.g., Tomasello et al., 2005), and precursors of generic codes of behavior (more local norms) could arise from these. Once the capacities allowing individuals to align with each other have evolved (and developed), group-level alignment (e.g., parochialism, common values and ways of doing things) can evolve, potentially as a result of pressures such as inter-group competition and cultural group-selection (Boyd and Richerson, 2002; Henrich, 2004). This account of alignment with others via empathy and other-regarding concerns, as well as an alignment with the group via normativity, can provide a fresh perspective on and thus contribute importantly to our understanding of humans’ ultrasociality.

## Conflict of Interest Statement

The authors declare that the research was conducted in the absence of any commercial or financial relationships that could be construed as a potential conflict of interest.

## Footnotes

1 The suggestion from experimental economics is that people should be rational maximizers, namely that they should be interested only in outcomes that affect themselves. Sensitivity to outcomes for others is referred to as an other-regarding preference. For example, giving up money in a dictator game is a departure from rational self-interested maximization (Camerer and Thaler, 1995).

2 Note that such modulation of empathic concern – and indeed all empathic concern – additionally requires the ability to regulate one’s emotions, which allows one to modulate and regulate one’s emotional response as appropriate. We will not deal with this vast topic here but the interested reader is referred to the theoretical and empirical work of Eisenberg and colleagues (Eisenberg and Fabes, 1990, 1992; Eisenberg, 2000; Eisenberg et al., 2006; Eisenberg and Eggum, 2009; see also Koski and Sterck, 2009, for a discussion of the emotion regulation capacities of chimpanzees).

3 This is not to say that there is not a close link between moral knowledge and moral action or using one’s moral knowledge (but see Blasi, 1983, for the intricacy of this relation).

4 Chimpanzees and bonobos do not reject unfair offers when tested with the ultimatum game (Jensen et al., 2007a; Kaiser et al., 2012; Proctor et al., 2013), though some non-human primates sometimes reject food when a partner receives better (Brosnan and de Waal, 2003; though see, for example, Bräuer et al., 2009).
